# Developing Advanced Chimeric Cell Therapy for Duchenne Muscular Dystrophy

**DOI:** 10.3390/ijms252010947

**Published:** 2024-10-11

**Authors:** Katarzyna Budzynska, Katarzyna T. Bozyk, Klaudia Jarosinska, Anna Ziemiecka, Krzysztof Siemionow, Maria Siemionow

**Affiliations:** 1 Dystrogen Therapeutics Technology Polska sp. z o.o., 00-777 Warsaw, Poland; kat.budzynska@gmail.com (K.B.); bozyk@dystrogen.com (K.T.B.); kaminska@dystrogen.com (K.J.); cieciuch@dystrogen.com (A.Z.); siemiok@dystrogen.com (K.S.); 2 Department of Orthopaedics, University of Illinois at Chicago, Chicago, IL 60607, USA; 3 Chair and Department of Traumatology, Orthopaedics, and Surgery of the Hand, Poznan University of Medical Sciences, 61-545 Poznan, Poland

**Keywords:** dystrophin-expressing chimeric (DEC) cells, DEC therapy, Duchenne muscular dystrophy, advanced therapy medicinal product, cell fusion

## Abstract

Duchenne Muscular Dystrophy (DMD) is a lethal, X-linked disorder leading to muscle degeneration and premature death due to cardiopulmonary complications. Currently, there is no cure for DMD. We previously confirmed the efficacy of human Dystrophin-Expressing Chimeric (DEC) cells created via the fusion of myoblasts from normal and DMD-affected donors. The current study aimed to optimize the development of DEC therapy via the polyethylene glycol (PEG)-mediated fusion protocol of human myoblasts derived from normal, unrelated donors. The optimization of cell fusion assessed different factors influencing fusion efficacy, including myoblast passage number, the efficacy of PKH myoblast staining, the ratio of the single-stained myoblasts in the MIX, and PEG administration time. Additionally, the effect of PEG fusion procedure on cell viability was assessed. A correlation was found between the number of cells used for PKH staining and staining efficacy. Furthermore, the ratio of single-stained myoblasts in the MIX and PEG administration time correlated with fusion efficacy. There was no correlation found between the myoblast passage number and fusion efficacy. This study successfully optimized the myoblast fusion protocol for creation of human DEC cells, introducing DEC as a new Advanced Therapy Medicinal Product (ATMP) for DMD patients.

## 1. Introduction

Duchenne Muscular Dystrophy (DMD) is the most severe type of inherited neuromuscular disorder, primarily affecting males with a frequency of 1 in 5000 live births [1,2,3,4]. DMD is characterized by progressive muscle degeneration associated with increased levels of fibrosis and inflammation, which consequently leads to premature death due to cardiopulmonary complications [5,6,7]. 

Currently, there is no effective or curative treatment available for DMD patients. Steroids remain the gold standard for symptom management and improving the quality of patients’ lives; however, they do not alter the progression of the disease [8,9]. Although gene therapies show promise, their applicability in DMD patients is limited by the types of genetic mutations [10]. Additionally, gene therapies carry known risks, such as off-target mutations and immune responses to the viral vectors used for the delivery of the therapeutic genes [11,12,13,14]. Alternatively, cell-based strategies offer the potential to treat a broader population of DMD patients, regardless of specific genetic mutations [15,16,17,18,19]. However, these approaches also face significant limitations in clinical applications [20,21]. Specifically, human myoblast transplantation is hindered by low cell engraftment, the insufficient migration of cells to the recipient muscles, and the toxicity associated with immunosuppression to prevent cell rejection [22]. Muscle stem cells (satellite cells) are considered a promising alternative due to their self-renewal capacity but only a limited number of satellite cells can be isolated from the dystrophic muscles [16]. Moreover, the intramuscular administration of cells is associated with high cell loss, and intravenous delivery raises safety concerns due to the microthrombi formation [16,23]. 

Techniques for cell manipulation, such as cell fusion, provide valuable tools for addressing these challenges. Several established methods of cell fusion include polyethylene glycol (PEG)-mediated fusion, electrofusion, and virus-induced cell fusion [24,25,26]. However, each method presents certain limitations in the practical applications [27]. Based on the need for innovative treatment strategies for DMD and our experience in developing stem cell therapies in regenerative medicine and reconstructive transplantation [28], we introduced a novel therapy of Dystrophin-Expressing Chimeric (DEC) cells with the potential to halt the progression of the disease. The creation of DEC cells is based on the ex vivo fusion of human myoblasts from normal and DMD-affected donors [29]. Preclinical studies testing the efficacy of human DEC therapy confirmed a long-term increase in dystrophin expression with improved muscle function and reduced pathological changes in the skeletal muscles following the intraosseous administration of DEC cells to the *mdx*/*scid* mouse model of DMD [30]. Furthermore, the safety and preferential long-term biodistribution of DEC cells to the DMD-affected target organs, including heart, diaphragm, and gastrocnemius muscles, were confirmed [31]. These encouraging findings led to the first-in-human study which tested the safety and preliminary efficacy of a single dose of DT-DEC01 therapy in three DMD patients up to 24 months following the systemic-intraosseous administration [32]. The positive outcomes of the pilot clinical study are encouraging; therefore, further assessment is intended to optimize DEC fusion protocol, aiming to introduce DEC therapy as a novel Advanced Therapy Medicinal Product (ATMP) and justify its application for larger scale clinical studies.

Advanced Therapy Medicinal Products involve substantially manipulated genes, somatic cells, or tissue-engineered products intended for human use [33,34]. These products are strictly regulated by the European Commission to ensure patient safety and clinical efficacy [35]. However, despite their potential to revolutionize the treatment of previously incurable diseases, ATMPs face significant challenges in the development phase and commercialization procedures [36]. 

The goal of this study was to optimize the development of human DEC cells created via the PEG-mediated fusion of human myoblasts derived from the unrelated donors to introduce human DEC therapy as a novel ATMP for clinical applications in DMD patients. The following factors were analyzed in the context of fusion efficacy: the number of myoblasts passages, the efficacy of myoblast staining with PKH26 and PKH67 dyes, the proportion of single-stained cells used for the fusion, the time of PEG addition and the effect of fusion procedure and PEG administration time on the cell viability. This study optimized the PEG-mediated myoblast fusion protocol for the manufacturing of the human chimeric cells, introducing DEC as a novel ATMP for treatment of DMD.

## 2. Results

**Figure 1:**
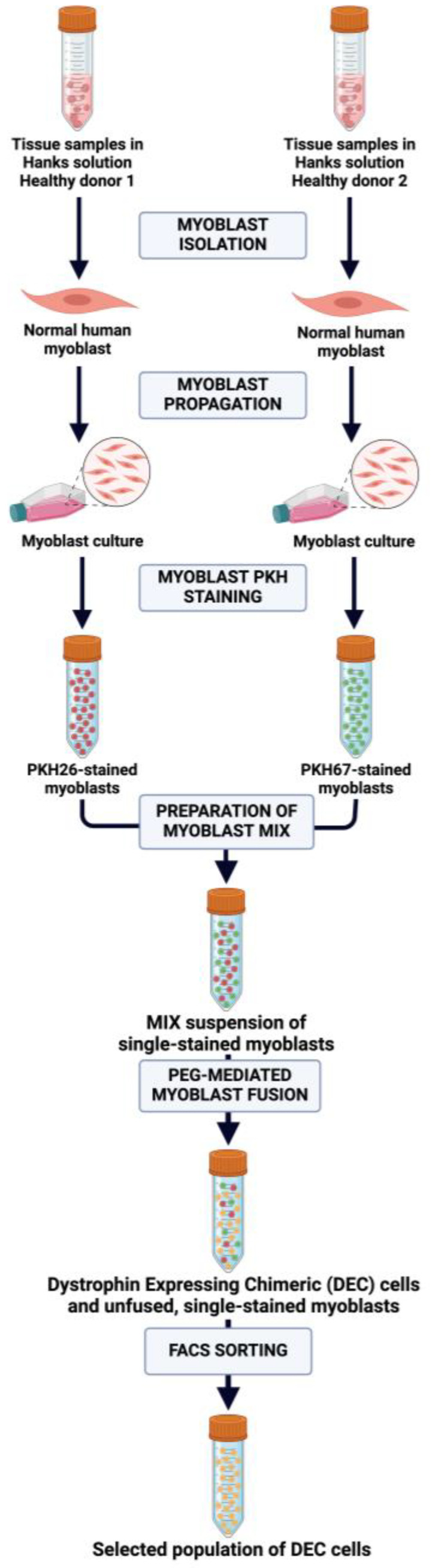
Diagram of ex vivo polyethylene glycol (PEG)-mediated myoblast fusion for creation of human Dystrophin-Expressing Chimeric (DEC) cells. Muscle tissue samples acquired from healthy, unrelated donors were collected in Hanks’ solution. Furthermore, the specimens were subjected to myoblast isolation and propagation, followed by myoblast staining with PKH26 (red) and PKH67 (green) fluorescent dyes. Two single-stained cell lines were mixed in one suspension (MIX) and fused in PEG for creation of human DEC cells, positive for both PKH26 and PKH67 dyes (orange). After fusion, from the cell suspension containing both single-stained and double-stained cells, the double-stained DEC cells (orange) were sorted to obtain a selected population of DEC. The figure was created with BioRender.com.

The creation of human DEC cells through the PEG-mediated fusion of PKH single-stained myoblasts from healthy, unrelated donors is presented in Figure 1.

### 2.1. Confirmation of the Creation of High-Viability Human DEC Cells via Ex Vivo PEG-Mediated Fusion

**Figure 2:**
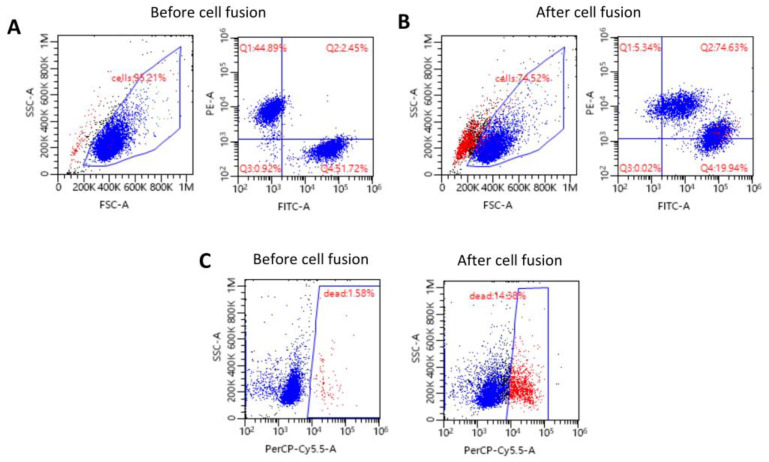
Confirmation of creation of DEC cells derived from normal, healthy donors. Representative flow cytometry dot plots confirm the cell fusion of human myoblasts stained with PKH26 or PKH67 dyes. The first row shows the following: (**A**) The MIX of single-stained PKH26 and PKH67 myoblast cell lines before the fusion procedure. In the SSC-A/FSC-A plot, the gate indicates the myoblast population based on the size and granularity (blue), while other objects represent the debris (red). The PE-A/FITC-A plot presents the populations of single-stained cells with PKH26 (Q1) and PKH67 (Q4) dyes. (**B**) Myoblasts after PEG-mediated fusion. The SSC-A/FSC-A plot presents the gating strategy to identify the cell population (gated, blue) and debris (red). The PE-A/FITC-A plot demonstrates fusion efficacy (Q2), with double-positive PKH26/PKH67 DEC cells, confirming the successful creation of human DEC cells. (**C**) The second row shows SSC-A/Per-Cy5.5-A plots indicating cell viability before (left image) and after (right image) PEG-mediated fusion. The gate indicates the population of dead cells visualized with 7-AAD dye (red), while the viable myoblasts are shown in blue.

The creation of human DEC cells by ex vivo PEG-mediated myoblast fusion derived from normal unrelated human donors was confirmed by fluorescence-activated cell sorting (FACS) (Figure 2A,B). Before fusion initiation, the FACS analysis of the MIX cell suspension confirmed 44.89% of single-stained myoblasts with PKH26 dye (Figure 2A) and 51.72% of single-stained myoblasts with PKH67 dye (Figure 2A). After the fusion procedure, the presence of the double-stained PKH26/PKH67 cells determined by FACS revealed a fusion efficacy of 74.63% (Figure 2B), confirming the successful creation of human DEC cells from the unrelated myoblast donors. Furthermore, the DEC cell viability, assessed before and after fusion, was 98.42% and 85.62%, respectively (Figure 2C). 

### 2.2. Confirmation of the Correlation between PKH Staining Efficacy and the Number of Myoblasts Used for Staining

**Figure 3:**
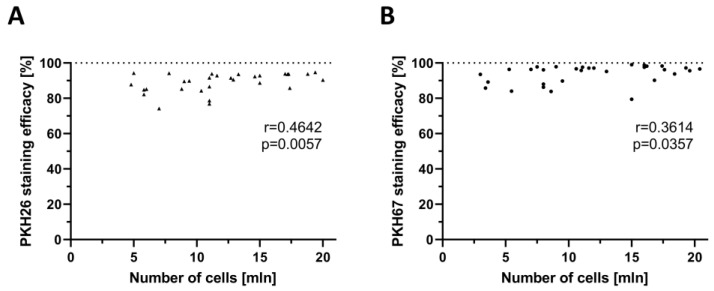
Correlation between the PKH staining efficacy and the number of myoblast cells used for staining. (**A**) Fair and significant correlation between the increase in the staining efficacy and the higher number of cells used for PKH26 staining (*r* = 0.4624). (**B**) Fair and significant correlation between the increase in the staining efficacy and the higher number of cells used for PKH67 staining (*r* = 0.3641). Each point illustrated on the graphs represents the staining efficacy value for a specific number of stained cells. Number of single cell staining with PKH26 dye, *n* = 36; number of single cell staining with PKH67 dye, *n* = 35. A nonparametric Spearman test was used to assess correlation.

A total of 71 single-staining procedures of human myoblasts were assessed to evaluate the correlation between PKH staining efficacy and the number of cells used for staining. A total of 36 of these procedures were performed with PKH26 (red) dye (Figure 3A) and 35 were performed with PKH67 (green) dye (Figure 3B). The efficacy of PKH26 staining ranged from 76.90% to 94.70% with the number of myoblasts used for the staining varying between 4.97 × 10^6^ to 20.00 × 10^6^ cells. Staining efficacy assessed for PKH67 varied between 79.48% and 99.00%, with myoblasts counts ranging from 2.99 × 10^6^ to 20.40 × 10^6^ cells. 

The assessment of correlation between staining efficacy and the number of myoblasts used for the staining revealed the following results: for PKH26, a correlation coefficient (*r*) of 0.4642 (*p* < 0.01), and for PKH67, a *r* of 0.3641 (*p* < 0.05), respectively. These findings confirmed a fair and significant correlation between the increase in PKH staining efficacy and the higher number of myoblasts used for the staining.

### 2.3. Confirmation of the Correlation between PKH Staining Efficacy and Fusion Efficacy

**Figure 4:**
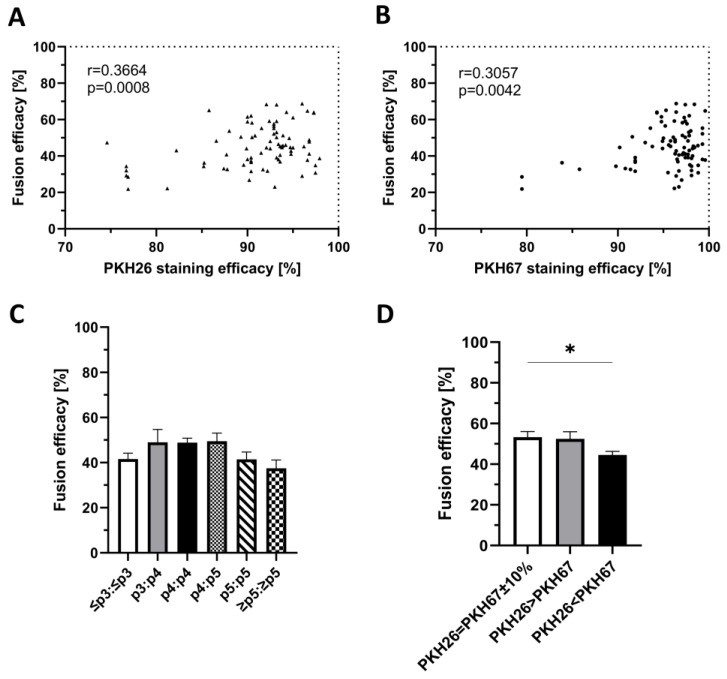
Correlation between fusion efficacy and PKH staining efficacy, number of myoblasts passages, and proportion of single-stained myoblasts in the MIX sample before fusion. (**A**,**B**) Fair and significant correlation between the increased efficacy of staining with (**A**) PKH26 (*r* = 0.3664) and (**B**) PKH67 (*r* = 0.3057) dyes and the increase in fusion efficacy. Each point illustrated on the graphs represents myoblast staining efficacy paired with the values of fusion efficacy; the analysis was obtained from an equal number of PKH26 and PKH67 myoblast stainings (*n* = 90) followed by 90 fusions. A parametric Pearson correlation test was used to assess the correlation. (**C**) No significant increase in fusion efficacy dependent on the number of passages for the myoblasts collection. A total of 87 PEG-mediated fusions were analyzed for myoblasts passages: ≤p3:≤p3 (*n* = 18); p3:p4 (*n* = 10); p4:p4 (*n* = 33); p4:p5 (*n* = 7); p5:p5 (*n* = 11); p≥5:p≥5 (*n* = 8). All data are presented as mean ± standard error of the mean (SEM). One-way ANOVA with Tukey’s multiple comparisons test was used to analyze the data. (**D**) A significant increase in the fusion efficacy at a 1:1 ratio of PKH26 and PKH67 single-stained myoblasts in the MIX (PKH26 = PKH67 ± 10%) when compared to the 0.75:1.0 (PKH26 < PKH67) ratio. Following numbers of fusions were evaluated for different proportions of single-stained myoblasts in the MIX (*n*): PKH26 = PKH67 ± 10%, *n* = 40; PKH26 > PKH67, *n* = 31; PKH67 > PKH26, *n* = 50. Total number of fusions, *n* = 121. All data presented as mean ± SEM. One-way ANOVA with Tukey’s multiple comparisons test was used to analyze the data. * *p* < 0.05.

A total of 90 myoblast fusion procedures were analyzed to assess the correlation between PKH staining efficacy and fusion efficacy, each preceded by the single staining of myoblasts with PKH26 (Figure 4A) and PKH67 (Figure 4B) dyes. The efficacy of PKH26 staining ranged from 74.60% to 97.92%, while the PKH67 staining efficacy varied from 79.48% to 99.60%. Fusion efficacy for both PKH stainings ranged from 21.90% to 68.80%. 

The assessment of correlation between the staining efficacy and fusion efficacy revealed correlation coefficient values for PKH26 (*r* = 0.3664, *p* < 0.001) and for PKH67 (*r* = 0.3057, *p* < 0.01), indicating a fair and significant correlation between the increase in PKH staining efficacy with the increase in the fusion efficacy. 

### 2.4. Confirmation of the Lack of Correlation between the Number of Myoblast Passages and Fusion Efficacy

A total of 87 fusion procedures were assessed to analyze the effect of myoblast passage number on fusion efficacy. The number of cell passages before cell fusion ranged from passage 3 to passage 6 (Figure 4C). The fusion efficacy ranged from 20.40% (fusion of myoblasts at passage 5) to 76.20% (fusion of myoblasts at passage 3 and passage 4). The mean values of fusion efficacy for the given passage numbers were as follows: for passages p≤3:p≤3, 41.55% ± 2.65%; for p3:p4, 48.96% ± 5.83%; for p4:p4, 48.85% ± 1.97; for p4:p5, 49.47% ± 3.65%; for p5:p5, 41.45% ± 3.28%; and for p≥5:p≥5, 37.48% ± 3.76. Fusion efficacy did not depend on the number of myoblasts passages.

### 2.5. Confirmation of the Correlation between the Ratio of Single-Stained Myoblasts before Cell Fusion and Fusion Efficacy

A total of 121 myoblast fusion procedures were assessed, involving three different cell count variants based on the ratio of the PKH26- and PKH67-stained myoblasts in the MIX suspension. The goal was to evaluate the effect of the ratio of the single-stained myoblasts before cell fusion on the fusion efficacy. The 1:1 ratio of the single-stained myoblasts (PKH26 = PKH67 ± 10%) was used in 40 fusion procedures, whereas the 1.5:1.0 ratio of PKH26:PKH67 single-stained myoblasts was used in 31 fusion procedures (PKH26 > PKH67), and the ratio of 0.75:1.0 of PKH26:PKH67 single-stained myoblasts was used in 50 fusion procedures (PKH26 < PKH67) (Figure 4D). The fusion efficacy was dependent on the ratio of the single-stained myoblasts in the MIX suspension before fusion. Mean fusion efficacy for the PKH26:PKH67-stained myoblast in the ratio of 1:1 (53.26% ± 2.81%) was significantly higher (*p* < 0.05) when compared to the 0.75:1.0 ratio (44.57% ± 1.75%) while no significant differences were found in the ratio of 1.5:1.0 in the MIX suspension (52.48% ± 3.47%). 

### 2.6. Confirmation of Shorter PEG Administration Time on Higher Myoblast Viability after Fusion

**Figure 5:**
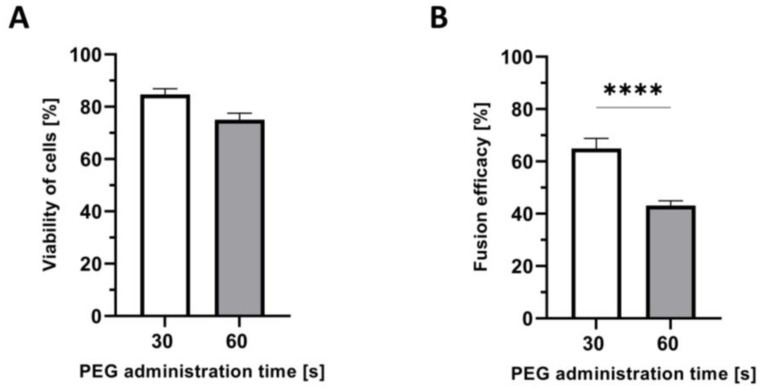
Correlation between the time of PEG administration, cell viability, and fusion efficacy. (**A**) Higher viability of myoblasts after the shorter time of PEG administration during the fusion procedure. Following number of fusions was assessed for different PEG addition times: 30 s, *n* = 15; 60 s, *n* = 45. (**B**) A significant increase in the fusion efficacy for the shorter time of PEG administration (30 s) when compared to the longer PEG addition time (60 s) during the fusion procedure. The following number of fusions were analyzed for different PEG addition times: 30 s, *n* = 10; 60 s, *n* = 45. Data presented as mean ± SEM. Unpaired two-tailed *t*-test was used to analyze the data. **** *p* < 0.0001.

A total number of 60 PEG-mediated fusion procedures were evaluated to analyze the effect of PEG administration time on myoblast viability. In 15 fusions, the PEG administration time was 30 s and in 45 fusions was 60 s (Figure 5A). Myoblast viability was higher (84.75% ± 2.16%) after shorter PEG addition time (30 s), when compared to the longer, 60 s, time of PEG administration (75.01% ± 2.53%). The difference was not significant but the 10% higher viability after shorter time of PEG administration presents an important trend and finding.

### 2.7. Confirmation of the Correlation between the Time of PEG Administration and Fusion Efficacy

A total of 55 PEG-mediated fusion procedures were assessed for the evaluation of the effect of PEG administration time on the fusion efficacy. In 10 fusion procedures, the time of PEG administration was 30 s, and in 45 fusions, it was 60 s (Figure 5B). The mean fusion efficacy of 64.92% ± 3.86% for the shorter PEG administration time (30 s) was significantly higher (*p* < 0.0001) when compared to the longer, 60 s, time of PEG addition (43.11% ± 1.87%). 

## 3. Discussion 

DMD is a progressive, muscle-wasting disease that leads to death in adolescence [1,37]. The current standard of care involves supportive steroid therapy, which helps reduce symptoms and improve patients’ quality of life [38]. However, steroids do not alter the progression of DMD and are associated with significant long-term side effects, including osteoporosis, growth retardation, glucose intolerance, and increased infection risks [39]. Therefore, there is a critical need for alternative therapeutic strategies represented by the ATMPs to more effectively address and manage the DMD progression and the symptoms.

ATMPs consist of medicinal treatments involving substantially manipulated genes, somatic cells or tissue-engineered products for human use [34]. The classification criteria for ATMPs are rigorously regulated by the European Commission under Regulation (EC) No. 1394/2007 and Directive 2001/83/EC to ensure patient safety and the clinical efficacy of the products [36]. The strict guidelines and requirements are designed to provide innovative strategies with promising potential for the management and treatment of otherwise incurable diseases. However, the manufacturing processes of ATMPs present challenges, particularly in terms of complexity of the development strategies, commercialization to enhance the feasibility at larger scales, and standardization to ensure compliance with good manufacturing practices (GMPs) [33,40]. 

In recent years, several gene- [11,41,42,43,44] and cell-based [15,16,45,46] therapies have been explored as potential treatments for DMD, aiming to restore dystrophin levels in the affected muscles [5,47]. While stem cell-based therapies offer a promising alternative, their routine application is limited by significant challenges [20,21,48]. Studies assessing myoblast and mesenchymal stem cells transplantation in DMD patients demonstrated the safety, but were challenged by the limited cell engraftment, low efficacy, and the need for supportive immunosuppression to prevent rejection [22,49,50,51,52,53]. However, encouraged by the potential of stem cells to alter disease progression and based on our experience with chimerism induction in transplantation [28], we developed a novel therapeutic approach for DMD by creating Dystrophin-Expressing Chimeric Cells of myoblast origin through the PEG-mediated cell fusion [29].

Cell fusion is a physiological process that occurs naturally in multicellular organisms during both developmental stages and throughout adulthood [54,55,56]. There are several methods of the ex vivo cell fusion reported, including PEG-mediated cell fusion, electrofusion and virus-induced fusion. However, each of these approaches presents significant challenges limiting their routine clinical application [27,57]. Electrofusion, which is based on the application of high-voltage electric pulses [58,59], demonstrates a high level of efficacy but requires advanced and expensive equipment as well as careful optimization due to potential cell damage from excessive electrical field strength [60,61]. The biological cell fusion involves noninfectious or inactivated viruses, such as the Sendai virus [62], but is limited by the length of the procedure, high cost, and potential for immunologic responses [63,64,65], which raises concerns about viral infection and further restricts its clinical applicability [26]. Although virus-induced fusion tends to result in high cell viability, cell health can also be compromised by activation of immune responses [26,65].

Therefore, considering the challenges associated with other fusion technologies, in our experimental studies, we applied the PEG-mediated cell fusion procedure, which is fast, inexpensive, and does not require additional equipment [66,67]. Moreover, this method is recognized for its safety and reproducibility [27]. The action of PEG is based on the aggregation of membrane vesicles, which leads to the close contact with the exclusion of volume and the removal of water in the contact area [24,68,69]. This technique is widely used in hybridoma technology to produce monoclonal antibodies through the fusion of B lymphocytes with the myeloma cells [70]. Furthermore, PEG-mediated cell fusion holds potential to establish the platform for different therapeutic applications. 

Based on our previous experience with the creation of different chimeric cell lines via PEG-mediated fusion [27,29], this study focused on optimizing the fusion procedure of creating human DEC cells as a potential novel ATMP for DMD patients. The ex vivo fusion of myoblasts using PEG is an established procedure that involves critical steps, including myoblast isolation, in vitro cell culture, myoblast staining with PKH26 (red) and PKH67 (green) membrane dyes, the preparation of a single-stained cell MIX suspension, PEG-mediated fusion, and subsequent FACS sorting. Since each of the presented steps may affect the quality of the created DEC cells, we analyzed the effect of each factor on the fusion efficacy and reproducibility with the goal of optimization of myoblast fusion for the DEC therapy.

This study confirmed a fair and significant correlation between the number of cells used for PKH staining and the staining efficacy for both the PKH26 and PKH67 dyes. Moreover, the higher staining efficacy was associated with a significant increase in the efficacy of the fusion procedure, which is potentially related to the effect of PKH dyes on cell membranes integrated in the fusion process. PKH dye incorporation into the membranes is stable, does not alter cell function, and provides a quantitative and histologic localization both in vitro and in vivo [71]. Moreover, increased staining efficacy improves cell tracking which correlates with detection of the chimeric cells and fusion efficacy. Interestingly, there was a lack of correlation between the myoblast passage number and fusion efficacy, indicating that the myoblast culture of the donor and recipient cell lines does not require the same number of the passages before cell fusion. This finding is important, since maintaining a synchronized cell culture routine by passaging the donor and recipient cells at the same time could present a challenge, considering the literature reports confirming different proliferation rates for cells derived from unrelated donors, which can be associated with multiple factors, including the heterogeneity of the cells [72,73]. Additionally, it was also verified that the ratio of the single-stained myoblasts in the cell MIX suspension before fusion affects fusion efficacy. For the PKH26 and PKH67 myoblast ratio of 1:1, the efficacy of fusion was found to be significantly higher compared to the 1.5:1.0 and 0.75:1.0 ratios. This indicates that maintaining an equal ratio of cells before fusion increases the likelihood of cell-to-cell contact and membrane fusion, which is important for producing a high yield of fused cells and is consistent with the existing literature reports [74,75]. Finally, the assessment of the effect of time of PEG administration on the cell viability revealed a 10% higher cell viability with a shorter time, when compared with longer time of PEG administration. These differences were not found to be significant, which correlates with the literature reports on different responses of cells to the PEG-exposure [76,77]. Since cell viability is crucial for the development of cell-based products, therefore a higher viability observed after shorter time of PEG administration provides an important finding for optimizing DEC products for clinical applications, where cell viability directly correlates with the product’s therapeutic effect [77]. The importance of duration of PEG administration was further confirmed by higher myoblast fusion efficacy observed after shorter time of PEG administration, providing another important factor for optimization during the development process of DEC therapy. 

This study confirmed the optimization and further standardization of PEG-mediated human myoblast fusion from normal, healthy unrelated donors. These findings will facilitate the standardization of the DEC manufacturing protocol on a large scale for clinical applications. As a result, human DEC therapy can be introduced as a new chimeric cell-based ATMP for large-scale clinical applications in DMD patients. 

## 4. Materials and Methods

### 4.1. Myoblast Isolation 

Remnants of muscle tissue were harvested from 27 normal, healthy donors after ACL surgery. The procedures described in this section received approval from the Bioethical Committee of Poznan University of Medical Sciences (permission number: 672/18) and complied with Good Laboratory Practice standards. Human myoblast preparation and isolation was performed according to our established protocol and literature reports, described previously in detail [73,78,79]. Briefly, the remnants of muscle tissue were transported to the R&D laboratory in the Hanks’ Balanced Salt Solution (HBSS, Nordmark Pharma GmbH, Uetersen, Germany). After discarding the connective and adipose tissue, the skeletal muscle tissue was minced, digested in 0.454 U/mL collagenase (Nordmark Pharma GmbH, Uetersen, Germany) for 45 min, filtered through a 70 µm mesh, and then centrifuged. The resultant cell pellet was resuspended in the culture medium of Dulbecco’s Modified Eagle Medium (DMEM, HyClone Laboratories, Logan, UT, USA) supplemented with 1% L-Alanyl-L-Glutamine (Biological Industries, Cromwell, CT, USA), 20% fetal bovine serum (FBS, Lonza Clonetics, Mapleton, IL, USA), 1% antibiotic–antimycotic (Gibco-ThermoFisher, Waltham, MA, USA), and 12 ng/mL of human basic fibroblast growth factor (hBFGF, Bio-Techne, Minneapolis, MN, USA), and cultured in T25 flasks coated with the mesenchymal stem cell (MSC) attachment solution (Genos, San Francisco, CA, USA).

### 4.2. Myoblast Propagation and Passage 

After the isolation of myoblasts from muscle tissue specimens, the cells derived from each donor were propagated to obtain a sufficient cell number to perform cell fusion. The myoblasts were propagated in culture media supplemented with 6 ng/mL hBFGF (Bio-Techne, Minneapolis, MN, USA). Upon reaching 60% to 70% confluence, myoblasts were passaged and harvested using TrypLE TE Select (Gibco-ThermoFuisher, Waltham, MA, USA). For the fusion procedure, human myoblasts were collected between the passage 3 and passage 6. Six groups were established according to the number of passages performed on both cell lines of the myoblasts used for fusion: p≤3:p≤3; p3:p4; p4:p4; p4:p5; p5:p5; p≥5:p≥5.

### 4.3. Myoblast Staining with PKH26 and PKH67 Dyes

After assessing the number of myoblasts after propagation using the ADAM™ cell counter (NanoEntek, Waltham, MA, USA) and dedicated commercial propidium iodide-based dyes (NanoEntek, Waltham, MA, USA), the isolated cells were washed in serum-free DMEM media supplemented with 1% antibiotics. Myoblasts from each donor were stained with the fluorescent dyes, PKH26 (red) or PKH67 (green) (Sigma-Aldrich, Saint Louis, MO, USA), using one dye per donor to ensure accurate myoblast tracking. The number of cells subjected to staining depended on the yield of the cell harvest before each fusion, with a minimum number of 2.0 × 10^6^ and maximum number of 20.5 × 10^6^ cells collected per single staining. Following the manufacturer’s instructions, each parent cell pellet was suspended in 1 mL of Diluent C (Sigma-Aldrich, Saint Louis, MO, USA) and mixed with 4 µL of either PKH26 or PKH67 dye also suspended in 1 mL of Diluent C. The myoblasts were then incubated for 5 min at room temperature (RT). The procedure was stopped by adding 1% bovine serum albumin (BSA, Miltenyi Biotec, Gaithersburg, MD, USA). The stained cells were initially washed in culture media and subsequently in serum-free DMEM culture media supplemented with 1% antibiotics.

### 4.4. Creation of Human DEC Cells via PEG-Mediated Cell Fusion Procedure and Cell Sorting

In total, 121 fusions were performed, each involving myoblasts from two different myoblast cell lines, which were obtained from unrelated, randomly selected donors. The number of cells for each donor was determined, and the volume of cell suspensions was adjusted with the intention to obtain MIX of cells with 1:1 cell count ratio. Furthermore, the proportion of cells in the MIX sample was verified by the FACS analysis. Three different proportions of the myoblasts single-stained with PKH26 and PKH67 dyes in the MIX suspension were identified: PKH26 = PKH67 ± 10% (PKH26:PKH67 ratio 1:1), PKH26 > PKH67 (PKH26:PKH67 ratio 1.5:1.0), and PKH26 < PKH67 (PKH26:PKH67 ratio 0.75:1.0) The fusion procedure was initiated by adding the PEG 4000 (Merck, Rahway, NJ, USA) solution containing 20% dimethyl sulfoxide (DMSO, WAK-Chemie Medical GmbH, Steinbach, Germany) with treatment durations of 30 s and 60 s, respectively. After fusion, the cell sorting procedure was preceded by a single wash of the cells and cells suspension in the MACS GMP PBS/MgCl_2_ sorting buffer (Miltenyi Biotec, Gaithersburg, MD, USA) supplemented with human albumin (Alburex20, CSL Behring, King of Prussia, PA, USA) to achieve a final concentration of 0.5% and MACS GMP Tytonase 1:20 (20 × Stock solution, Miltenyi Biotec, Gaithersburg, MD, USA). Next, the double-positive PKH26/PKH67 DEC cells were selected using the FACS MACSQuant Tyto (Miltenyi Biotec, Gaithersburg, MD, USA).

### 4.5. Cytometric Analysis of Fusion Efficacy and Cell Viability

The efficacy of the PEG-mediated fusion procedure for creation of human DEC cells and the viability of DEC cells was assessed by FACS before and after the fusion. To estimate the efficacy of cell fusion, cytometric settings were adjusted on the MIX sample (single-stained myoblasts before fusion). Gating strategy was used to measure fusion efficacy, defined as the percentage of the double positive cells (showing both the PKH26 and PKH67 signals) in the Q2 quadrant of PT-A/FITC-A plot. Cell viability was assessed in the MIX sample before cell fusion and in the cell suspension sample after fusion and was determined using 7-actinoaminomycin D (7-AAD, 1:5 working dilution; BD Biosciences, Franklin Lakes, NJ, USA). Each sample, containing 0.1 × 10^6^ cells, was incubated for 10 min at RT with the cell viability solution 7-AAD and next was assessed by flow cytometry (BriCyte E6, Mindray, Mahwah, NJ, USA) for the presence of the PerCP-Cy5.5 signal.

### 4.6. Statistical Analysis

Data are presented as the mean ± standard error of the mean (SEM). GraphPad Prism (ver. 9.5.0 and 10.0.0, RRID: SCR_002798, Dotmatics, Boston, MA, USA) software was used for statistical analyses. The comparison between the number of myoblasts passages before cell fusion and fusion efficacy, as well as the comparison between the ratio of PKH26- and PKH67-stained myoblasts in the MIX and fusion efficacy was analyzed using the ordinary one-way analysis of variance (ANOVA) with Tukey’s multiple comparisons test. A parametric Pearson correlation test was used to assess the correlation between PKH staining efficacy and fusion efficacy, whereas a nonparametric Spearman test was used to assess the correlation between PKH staining efficacy and the number of cells used for the staining. The correlation was considered strong with a *r* of ≥0.8, moderately strong with a *r* between 0.6 and 0.8, and fair with a *r* between 0.3 and 0.6 [80]. For the comparison of timings of PEG addition, fusion efficacy, and cell viability, an unpaired two-tailed *t*-test was used. Results were considered significant at *p* < 0.05.

## 5. Conclusions

This study confirmed the optimization and further standardization of the PEG-mediated human myoblast fusion protocol and established the feasibility and reproducibility of DEC cell creation. Several factors correlated with increased myoblast fusion efficacy, including the PKH staining efficacy, the ratio of single-stained myoblasts in the MIX, and PEG administration time. No correlation was found between fusion efficacy and the number of myoblasts passages before cell fusion. These findings support the further standardization of the DEC cell manufacturing protocols. Consequently, human DEC therapy can be introduced as a new ATMP for large-scale clinical applications in DMD patients. 

## Data Availability

The original contributions presented in the study are included in the article, further inquiries can be directed to the corresponding author.
